# The impact of the Great Recession on mental health and its inequalities: the case of a Southern European region, 1997–2013

**DOI:** 10.1186/s12939-015-0283-7

**Published:** 2016-01-26

**Authors:** Amaia Bacigalupe, Santiago Esnaola, Unai Martín

**Affiliations:** Department of Sociology 2, University of the Basque Country (UPV/EHU), Barrio Sarriena s/n. 48940, Leioa, Spain; Department of Health, Basque Government, Donostia-San Sebastian 1. 01010, Vitoria-Gasteiz, Spain

**Keywords:** Economic recession, Spain, Socioeconomic factors, Mental health, Unemployment

## Abstract

**Background:**

Numerous studies have shown that macroeconomic changes have a great influence on health, prompting different concerns in recent literature about the effects of the current recession. The objetive of the study was to assess the changes in the mental health of the working-age population in the Basque Country (Spain) and its social inequalities following the onset of the 2008 recession, with special focus on the role of unemployment.

**Methods:**

Repeated cross-sectional study on the population aged 16–64, using four Basque Health Surveys (1997–2013). Age-adjusted prevalences of poor mental health and incremental prevalence ratios (working status and social class adjusted) between years were calculated. Absolute/relative measures of social inequalities were also calculated.

**Results:**

From 2008, there was a clear deterioration in the mental health, especially among men. Neither changes in employment status nor social class accounted for these changes. In men, the deterioration affected all working status categories, except the retired but significant changes occurred only among the employed. In women, poor mental health significantly increased among the unemployed. Students were also especially affected. Relative inequalities increased only in men.

**Conclusions:**

The Great Recession is being accompanied by adverse effects on mental health, which cannot be fully explained by the increase of unemployment. Public health professionals should closely monitor the medium and long-term effects of the crisis as these may emerge only many years after the onset of recessions.

## Background

Since the onset of the financial crisis in 2007, Western countries have been going through a deep recession with huge impacts not only in the economic sphere, but also in the social, cultural and political arenas. Many structural and intermediary determinants of health have undergone a profound transformation, as can be seen in the escalating mass unemployment, increasing flexibility and non-standardized forms of employment, cuts in wages and other benefits, and growing poverty or social inequalities [1]. All these effects have been especially serious in southern European countries, where the International Monetary Fund and the European institutions have imposed strict austerity measures, implementing large-scale cuts and a generalized dismantling of the public sector [2].

Numerous studies have shown that macroeconomic changes have a great influence on the health of populations, prompting different concerns in recent literature about the effects of the current recession [3, 4], which reflect different views about the relationship between crises and health. Some analyses, primarily based on mortality data, show an improvement in population health during economic downturns [5]. Others, however, insist that the aggregated relationships cannot be directly translated to the individual level, where there is conclusive evidence that socioeconomically disadvantaged populations suffer higher ill health and mortality [6]. In line with this view, some other studies have demonstrated that when economic conditions worsen during crises, poor physical and mental health, and mortality tend to rise [3] and health inequalities can increase [7]. It is striking, then, that the impact of this economic crisis on social inequalities in health has received so little attention among researchers [4, 8].

Since the onset of the current crisis, the country-specific analyses carried out on mental health have focused on changes in prevalence of depression and anxiety symptoms, suicide related mortality rates, incidence of suicidal ideation and prevalence of perceived mental health, showing either a generalised deterioration across the population as a whole or in specific groups [9], especially in Spain [10–14], Italy [15, 16], Greece [17–20] and the UK [21]. A recent comparative study in Europe has shown that the increase in depression was noticeable in countries that have been strongly hit by the economic crisis, such as Cyprus and Spain [22].

In Spain, an increase in the frequency of diagnosed mental disorders in primary care has been reported [12], as well as short-term mental health risks [13], anxious and depressive symptoms in men [14] and suicidal ideation [10] while mixed results have been described for suicide related mortality [11, 23]. An important limitation of these studies is that most of them compare only two time-points, and therefore have a limited temporal perspective, affecting the ability to assess the changes that have occurred in addition to pre-crisis trends. Moreover, data on the impact on mental health inequalities is almost inexistent. The scarce data available shows an increase in educational level inequalities in diagnosed mental disorders [12] and inequalities in anxious and depressive symptoms by social class and education in men [14].

The relationship between economic crises and health or health inequalities can be attributed to a number of interlinked factors. Among these, rising unemployment seems to be a clear mediator leading to increased mortality from external causes, risky coping behaviours, or worsening of self-rated health during crises [21, 24, 25]. Mental health is known to be affected by unemployment due to the deterioration in self-esteem and pessimism about the future [26] as well as to the loss of income that hinders the access to different resources and to healthy lifestyles [27]. Interestingly, it appears that workers who keep their jobs during a recession are not immune to the adverse health effects of the crisis [28, 29] due to the fear and insecurity of a possible job loss, and increased workload [30, 31]. Among the employed, those in nonstandard jobs such as involuntary part-time or temporary have also shown higher levels of distress [22]. Regarding gender, different studies have shown that men are at increased risk of depression and other mental health disorders during crises [14, 21, 22], which could be related to women’s greater involvement in family responsibilities and consequent higher probability of finding alternative rewards in their family caregiver role when loosing their job [32]. Strong indicators of a more negative health effect due to unemployment have also been identified for manual workers, compared to other occupational categories [33].

Since 2008, Spain has been severely affected by the global crisis, with unemployment rates jumping from 7.9 and 11.9 % in 2008 to 26.7 and 27.3 % in 2013 in men and women respectively, according to the Spanish National institute for Statistics. Households’ difficulties for making ends meet has also increased clearly, from 28.8 % in 2007 to 38.8 % in 2013, and income inequality indicators are at record levels for the EU. However, the economic and social consequences of the crisis as well as the policies adopted vary greatly in the different Spanish regions [34]. The Basque Country is in a relatively advantageous position, probably due to an economic structure which has been less vulnerable to the effects of the recession, and to a more highly developed social protection system compared to other regions. That could probably explain the weaker impact of the Great Recession on unemployment levels that reached 15.1 and 14.0 % in Basque men and women in 2013.

The aim of this paper is to assess the medium-term changes in population mental health and its social inequalities following the onset of the 2008 recession in the general working age population as well as in different subgroups of the Basque Country, with a special focus on the role played by unemployment in the observed changes, from 1997 to 2013.

## Methods

### Design, study population, and data sources

**Fig. 1:**
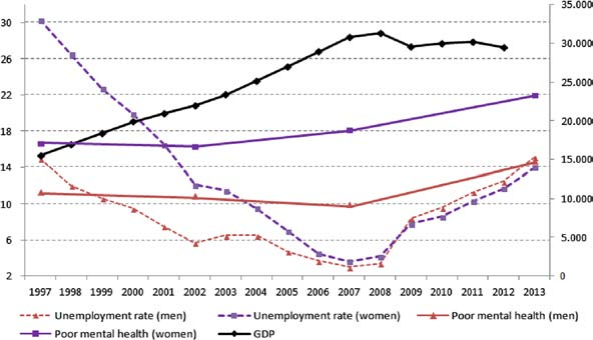
Gross Domestic Product (GDP), unemployment rate and age-standardized prevalence of poor mental health, by sex. Basque Country, Spain. 1997–2013

A repeated cross-sectional study was performed using data from the 1997, 2002, 2007 and 2013 rounds of the Health Survey of the Basque Country. All surveys were based on large random samples of private households, covering the non-institutionalized population. Data was collected by means of face-to-face interviews in the homes of the participants. Response rates for each of the surveys were 87, 86, 79 and 86 % respectively. The analysis was restricted to the population aged 16–64 years. In order to complement the socioeconomic context, data for annual GDP and unemployment rates (shown in Fig. 1) were obtained from the Quarterly Economic Accounts and the Labour Force Survey of the Basque Country, provided by the Basque Institute of Statistics.

### Variables

The dependent variable was poor mental health, derived from the Mental Health Inventory Scale (MHI-5), a five-question based subscale of the SF-36 used for detecting risk of depression and anxiety. The score for the MHI-5 was computed by adding up the scores of all question items. The cut-off value for identifying poor mental health was 52, in line with other studies [35]. The main independent variable was the survey year, which was used for creating three analysis periods: two pre-crisis (1997–2002; 2002–2007) and one crisis period (2007–2013). Working status distinguished between employed, unemployed, students, homemakers, retired and others. Social class was assigned according to the current or previous occupation of the interviewee or, if he or she had never worked, according to the occupation of the head of the household, following the Goldthorpe-oriented classification proposed by the Spanish Society of Epidemiology [36]. Five groups were identified from social class I -most advantaged- to social class V -most disadvantaged.

### Statistical analysis

Crude and age-standardised prevalences of poor mental health were calculated for each sex and survey year. For age standardization, the direct method was used based on the standard European population. To evaluate the impact of the crisis on poor mental health, we sought to determine if the underlying trend in poor mental health changed after the onset of the financial crisis (2007). This was made by calculating the incremental prevalence ratio (IPR) between contiguous survey years [37]. A change in the value of the incremental prevalence ratio between two time periods would suggest the presence of an effect (e.g., the economic crisis) added to the underlying trend in poor mental health. To estimate crude and adjusted IPR, we used Poisson regression models with robust error variance [38] and incremental coding of the year of the survey [39]. The contribution of unemployment to the changes in poor mental health was evaluated by comparing the age-adjusted IPRs with that adjusted by age and working status. IPRs were also calculated for subgroups according to age, working status and social class.

The magnitude of social class inequalities in poor mental health was measured, first, using the relative index of inequality (RII). Individuals were assigned a value between 0 and 1, representing the relative position of their social class in the social hierarchy and this value was related to poor mental health using a Poisson regression model with robust error variance. The RII is interpreted as the prevalence ratio between the most and the least deprived [40]. The Slope Index of Inequality (SII) was calculated using the previous regression model results, with the following formula: SII = exp(a + b) -exp(a), where a and b are the intercept and the coefficient corresponding to the RII respectively. The SII is interpreted as the absolute difference between the most and the least deprived. Changes in the RII were estimated by introducing in the regression model a product term of the variables corresponding to the year of the survey and the relative position of their social class. The impact of inequalities in poor mental health was measured using the absolute version of the population-attributable risk (PAR). Poisson regression-based prevalence ratios were applied to a hypothetical data matrix where all the individuals had the value of the highest social class, and the difference between the number of observed and estimated cases was calculated. All the analyses were separated for men and women, and were conducted using the SAS version 9.4.

## Results

A total of 20,231 participants were included in the analysis. In the later years of the survey, the participants were older, especially women, and the proportion of people in extreme social classes I and V increased (Table 1). Figure 1 shows that in both sexes, unemployment showed a U-shaped pattern, with a sharp descending trend reaching its minimum value in 2007 (3,0 % in men and 3,6 % in women) and a clear increase thereafter, reaching 15,1 and 14,0 % in men and women respectively. GDP followed the opposite trend, growing steadily until 2007, and decreasing slightly since then. Slight differences in the magnitude of unemployment were reported depending on the data source used (Table 1 uses Health Survey data & Fig. 1 uses Labour Force Survey data). Employed population decreased clearly from 2007 to 2013, both in men and women. Regarding inactive categories, a steady decrease of homemakers was seen among women, while the retired remained stable in men and went up among women. Student population decreased in both sexes. The prevalence of poor mental health was consistently higher in women than in men throughout the period (Table 1).

**Table 1:** Distribution (%) of the sample and prevalence (%) of poor mental health by sex and year of the survey. Basque Country, Spain. 1997-2013

	1997		2002		2007		2013	
	column %	Poor mental health (%)	column %	Poor mental health (%)	column %	Poor mental health (%)	column %	Poor mental health (%)
Men								
	*N* = 1.283		*N* = 3.103		*N* = 2.616		*N* = 2.597	
Total	100.0	11.4	100.0	11.0	100.0	10.0	100.0	14.7
Years of age								
16–24	22.2	6.6	18.0	8.5	13.6	7.6	11.7	10.9
25–34	21.3	9.8	26.2	10.1	25.3	8.7	20.8	10.8
35–44	23.3	12.9	21.3	10.2	23.2	9.2	25.6	16.2
45–54	18.2	13.2	19.0	13.9	18.4	12.7	23.2	18.5
55–65	15.0	16.5	15.6	12.9	19.4	11.9	18.6	14.6
Working status								
Employed	65.5	9.8	75.5	10.6	76.9	9.0	65.7	12.5
Unemployed	9.9	21.3	5.6	15.9	4.2	16.3	16.7	23.6
Homemaker	.	.	.	.	.	.	.	.
Student	14.5	7.5	11.4	6.3	9.4	6.1	9.5	10.7
Retired, others	10.2	18.4	7.5	18.3	9.5	19.4	8.1	19.0
Social class								
I	11.3	11.5	10.7	6.6	11.6	8.2	13.2	8.6
II	11.8	8.6	11.2	10.5	11.1	11.1	9.2	12.2
III	24.1	9.5	28.5	11.8	26.4	11.2	19.7	14.4
IV	42.5	12.0	40.2	10.7	41.6	8.3	48.3	15.5
V	10.3	16.7	9.3	15.4	9.2	15.4	9.7	22.1
Women								
	*N* = 1.645		*N* = 3.301		*N* = 2.855		*N* = 2.831	
Total	100.0	16.7	100.0	16.3	100.0	18.2	100.0	22.0
Years of age								
16–24	20.9	13.4	17.1	14.5	13.4	16.0	11.4	19.9
25–34	20.9	14.2	22.7	15.6	22.2	16.0	20.2	20.7
35–44	24.4	14.9	24.1	14.9	25.0	16.0	24.6	22.3
45–54	18.1	20.6	20.3	16.6	20.2	21.0	24.0	20.9
55–65	15.7	22.5	15.7	21.2	19.1	22.6	19.8	25.8
Working status								
Employed	34.5	14.5	46.9	15.1	57.2	16.4	55.1	18.8
Unemployed	11.4	18.1	6.3	15.7	5.2	20.0	13.0	31.4
Homemaker	38.7	18.5	31.4	17.7	22.8	22.6	17.4	25.9
Student	13.6	12.7	12.7	14.7	10.0	13.6	9.6	19.7
Retired, others	1.7	39.4	2.6	30.7	4.8	27.6	4.9	25.1
Social class								
I	11.0	10.9	11.4	12.1	10.3	12.1	12.6	18.8
II	10.2	15.4	11.2	14.4	12.1	15.4	10.6	15.5
III	25.7	13.5	31.5	16.1	28.8	17.2	26.5	20.2
IV	42.8	18.3	34.1	17.1	36.4	20.1	34.2	24.5
V	10.4	25.0	11.8	20.4	12.4	23.4	16.1	26.8

Table 2 shows that in the two pre-crisis periods, mental health in men improved slightly, although not significantly, and clearly worsened from 2007 to 2013 (IPR 2013/2007: 1.44 [95 % CI: 1.23–1.69]). In women, increased prevalence in poor mental health was noticeable from 2002, although the change was only significant from 2007 to 2013 and less pronounced than in men (IPR 2013/2007: 1.19 [95 % CI: 1.07–1.34]). Table 2 also shows that, for the crisis period, the significant increase in poor mental health was still apparent, although reduced, in both sexes after adjusting by working status. This was especially true for men but also for women (IPR men 2013/2007: 1.32 [95 % CI: 1.13–1.55]; IPR women 2013/2007: 1.18 [95 % CI: 1.05–1.32]). The subsequent addition of occupational social class as an adjustment variable to the model did not modify the observed relationships.

**Table 2:** Adjusted incremental prevalence ratios (IPR) and confidence interval (CI) of 95%, and age-adjusted IPR stratified by different social groups, according to sex and periods. Basque Country, Spain. 1997–2013

	2002/1997		2007/2002		2013/2007	
	IPR	CI 95%	IPR	CI 95%	IPR	CI 95%
Men						
Total population						
Age-adjusted	0.96	(0.79-1.16)	0.89	(0.76-1.04)	1.44	(1.23-1.69)
Age+ws^a^ adjusted	1.00	(0.83-1.21)	0.90	(0.77-1.05)	1.32	(1.13-1.55)
Age+ws+sc^b^ adjusted	1.00	(0.83-1.21)	0.90	(0.77-1.05)	1.34	(1.14-1.57)
Years of age^c^						
16-24	1.28	(0.74-2.23)	0.89	(0.56-1.42)	1.43	(0.86-2.39)
25-34	1.02	(0.65-1.60	0.87	(0.61-1.25)	1.24	(0.82-1.87)
35-44	0.79	(0.52-1.19)	0.91	(0.63-1.32)	1.73	(1.22-2.47)
45-54	1.05	(0.73-1.53)	0.90	(0.68-1.21)	1.46	(1.09-1.94)
55-64	0.78	(0.53-1.15)	0.93	(0.68-1.27)	1.23	(0.92-1.64)
Working status						
Employed	1.09	(0.86-1.39)	0.83	(0.69-1.01)	1.34	(1.10-1.64)
Unemployed	0.74	(0.44-1.24)	0.95	(0.53-1.72)	1.45	(0.88-2.40)
Student	0.84	(0.42-1.67)	0.97	(0.50-1.86)	1.76	(0.94-3.31)
Retired, others	0.95	(0.60-1.51)	1.14	(0.79-1.63)	0.95	(0.66-1.36)
Social Class						
I	0.55	(0.29-1.04)	1.24	(0.70-2.21)	1.01	(0.60-1.71)
II	1.22	(0.65-2.31)	1.05	(0.65-1.70)	1.10	(0.65-1.88)
III	1.23	(0.82-1.84)	0.92	(0.69-1.23)	1.28	(0.93-1.77)
IV	0.90	(0.68-1.19)	0.75	(0.59-0.97)	1.82	(1.43-2.32)
V	0.92	(0.55-1.54)	1.00	(0.66-1.54)	1.38	(0.91-2.11)
Women						
Total population						
Age-adjusted	0.97	(0.85-1.12)	1.11	(0.99-1.24)	1.19	(1.07-1.34)
Age+ws^a^ adjusted	1.00	(0.87-1.15)	1.13	(1.00-1.26)	1.18	(1.05-1.32)
Age+ws+sc^b^ adjusted	1.01	(0.88-1.16)	1.12	(1.00-1.25)	1.17	(1.04-1.31)
Years of age						
16-24	1.08	(0.75-1.55)	1.10	(0.80-1.51)	1.25	(0.88-1.77)
25-34	1.10	(0.80-1.51)	1.03	(0.78-1.36)	1.29	(0.96-1.74)
35-44	1.00	(0.74-1.35)	1.07	(0.82-1.40)	1.40	(1.08-1.81)
45-54	0.81	(0.61-1.06)	1.26	(1.02-1.56)	0.99	(0.81-1.22)
55-64	0.94	(0.71-1.25)	1.07	(0.87-1.32)	1.14	(0.94-1.38)
Working status						
Employed	1.04	(0.82-1.32)	1.07	(0.90-1.27)	1.12	(0.95-1.32)
Unemployed	0.85	(0.54-1.35)	1.25	(0.77-2.02)	1.56	(1.05-2.31)
Homemaker	0.94	(0.76-1.16)	1.25	(1.03-1.52)	1.15	(0.93-1.42)
Student	1.16	(0.74-1.81)	0.93	(0.63-1.38)	1.44	(0.94-2.20)
Retired,others	0.74	(0.42-1.30)	0.94	(0.63-1.41)	0.93	(0.64-1.35)
Social Class						
I	1.10	(0.64-1.92)	1.00	(0.65-1.55)	1.55	(1.04-2.33)
II	0.93	(0.59-1.46)	1.07	(0.74-1.56)	1.00	(0.67-1.49)
III	1.18	(0.88-1.58)	1.05	(0.85-1.30)	1.16	(0.92-1.46)
IV	0.93	(0.76-1.15)	1.16	(0.96-1.39)	1.21	(1.01-1.45)
V	0.83	(0.59-1.16)	1.08	(0.82-1.42)	1.13	(0.88-1.46)

^a^ Adjusted by age and working status; ^b^ Adjusted by age, working status and social class; ^c^Age-specific IPR

The stratification by age, working status and social class offers further insights to better understand the previous results: first, it shows that the increase in poor mental health during the period 2007–2013 was especially relevant among those aged 35–44, both in men (IPR 2013/2007: 1.73 [95 % CI: 1.22–2.47]) and women (IPR 2013/2007: 1.40 [95 % CI: 1.08–1.81]). Moreover, with regard to working status, it is interesting to note that from 2007 to 2013 poor mental health increased in all groups of men except the retired, although the increase was statistically significant only in those who were employed (IPR 2013/2007: 1.34 [95 % CI: 1.10–1.64]). Students and unemployed persons were also found to be more likely to report poor mental health in 2013 compared to 2007. Regarding social class, the increase in poor mental health was especially relevant among manual workers (IV and V). In women, however, the decline in mental health was evident among the unemployed in 2013 compared to 2007 (IPR 2013/2007 1.56 [95 % CI: 1.05–2.31]), although this tendency was somehow perceptible in the period 2002–2007 (IPR 2007/2002 1.25 [95 % CI: 0.77–2.02]). Deterioration in mental health was also found among students and, less clearly, among homemakers and the employed. Regarding social class, poor mental health was found to have increased in class IV, but especially among the better off (IPR 2013/2007: 1.55 [95 % CI: 1.04–2.33]).

**Fig. 2:**
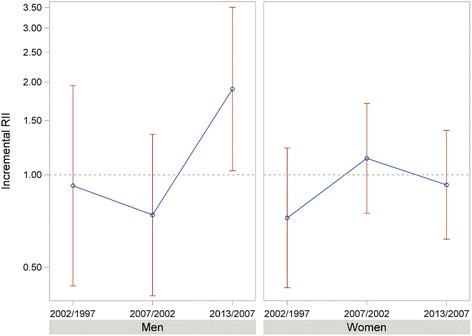
Changes in the relative index of inequality by social class of poor mental health. Basque Country, Spain. 1997–2013

Table 3 shows that social class inequalities were relevant throughout the period, with higher poor mental health among lower classes in both sexes. However, a different gender pattern was observed from 1997 on: in men, relative inequalities steadily decreased until they disappeared in 2007 (RII: 1.10 [95 % CI: 0.70–1.80]), then they rose significantly from 2007 to 2013 (RII 2013/2007: 1.90 [95 % CI: 1.03–3.05]) (Fig. 2). Meanwhile, absolute inequalities were almost nonexistent until 2007, but became relevant in 2013 (SII: 7.10 [95 % CI: 3.10–11.10]). The population attributable risk (PAR) revealed that in 2013 6.0 % of the total poor mental health prevalence in men (14.7 %) was attributable to social class inequalities. For women, no relevant changes in relative inequalities were observed for the period (RII 2013/2007: 0.93 [95 % CI: 0.62–1.39]), while a slightly upward trend in absolute inequalities was seen from 2007. The PAR showed that, in 2013, 3.0 percentage points out of the total poor mental health prevalence in women (22.0 %) were attributable to social class inequalities.

**Table 3:** Age-standardized prevalence (%), relative index of inequality (RII), absolute index of inequality (SII) and the absolute version of the population attributable risk (PAR) due to inequalities, of poor mental health by social class, sex, and year of the survey. Basque Country, Spain. 1997–2013

	1997		2002		2007		2013	
Men								
Total (%)	11.6		10.8		9.9		14.1	
Social class (%)								
I (highest)	12.7		6.5		8.4		9.1	
II	10.3		9.9		10.0		11.4	
III	9.3		11.8		11.0		13.3	
IV	12.2		10.2		8.3		14.5	
V (lowest)	14.8		13.3		15.2		17.1	
RII (CI 95 %)	*1.7*	(0.9–3.2)	1.6	(1.1–2.3)	1.1	(0.7–1.8)	2.2	(1.5–3.3)
SII (CI 95 %)	*2.8*	(−0.5–6.2)	3.2	(0.3–6.1)	0.8	(−1.6–3.1)	7.1	(3.1–11.1)
PAR	−0.7		4.4		1.7		6.0	
Women								
Total (%)	17.0		16.3		18.0		21.7	
Social class (%)								
I (highest)	11.1		11.1		12.7		18.0	
II	15.8		15.0		18.3		16.4	
III	14.0		16.3		16.3		19.6	
IV	19.9		17.1		19.9		22.4	
V (lowest)	25.3		22.0		22.7		26.5	
RII (CI 95 %)	*2.1*	(1.4–3.3)	1.6	(1.2–2.1)	1.8	(1.3–2.4)	1.7	(1.3–2.2)
SII (CI 95 %)	*7.4*	(2.0–12.9)	5.0	(1.3–8.7)	7.3	(2.5–12.0)	9.7	(3.7–15.7)
PAR	5.6		4.2		5.9		3.0

## Discussion

To our knowledge, this is one of the first population-based studies to analyse the impact of the Great Recession on mental health and its social inequalities in Southern Europe, to include a wide pre-crisis period and to analyse the specific role of unemployment and occupational social class in the observed changes. The results show that, since the onset of the economic crisis, there has been a significant deterioration in the mental health in the Basque Country, especially clear among men. However, this could not be attributed only to the increase of unemployed population during the study period. The stratified analysis showed, first, that the decline in mental health was especially relevant in the population aged 35–44. Second, mental health worsened in all male working status categories, except the retired, but significant changes were only observed among the employed. Students were also one of the groups showing the greatest deterioration in mental health among men. On the other hand, in women poor mental health only increased significantly among the unemployed from the onset of the crisis, although students were also affected. Retired women did not appear to suffer any special impact. Regarding occupational social class inequalities in mental health, these were relevant throughout the period, but with gender differences. In men, after a steady decrease of relative inequalities until 2007, they rose significantly from the onset of the crisis. On the contrary, no clear changes occurred among women.

Various limitations should be acknowledged before further discussion of these findings. First, the MHI-5 scale only covers affective symptoms and mood, but does not include impact on day-to-day functioning. In spite of this, it remains a useful instrument for studying trends in population mental health [41]. Second, the use of a repeated cross-sectional analysis limited the analysis of the effect of changes in individual employment status on health, which would have been possible if a longitudinal analysis had been used. Third, it is difficult to attribute changes in mental health directly to the economic crisis, as there were other parallel phenomena occurring in the same period. However, after studying a 21 years period, the results clearly show that the observed changes after the onset of the crisis did not occur in the previous years, this being especially evident for men. Moreover, we have explored only the role played by working status in the changes in mental health, but quality of employment, job insecurity and unemployment benefits have also proved important to our understanding of the effect of the economic crises on mental health [42]. Unfortunately, these variables were unavailable for all the surveys.

The results provide a more up-to-date picture of the impact of the current economic crisis on population mental health, confirming a generalized deterioration [4, 12], especially among men [14, 21] and in Southern European countries where the crisis has been especially deep [22]. Studies focusing on the increased suicide mortality and suicide attempts in Europe are also in line with these results [10, 11, 15, 18, 24].

In contrast with our results, a number of studies have found an increase in mental health problems during the Great Recession only among women [43] or no increase for either sex [29]. As described elsewhere, the reasons for these gender differences are not yet clear but may be partly attributable to the different situation of women in the labour market, the conditions in which women sometimes are forced into the labor market to address household income loss, differences between men and women as they cope with adapting to unemployment, or differences in the nature of the recession itself across countries [22, 43, 44].

Our results describe the weak role of changes in working status for explaining the decline of mental health after the onset of the crisis. This contrasts with other studies in Southern Europe, where the observed increase in poor mental health or suicide attempts is clearly attributed to the changes in the composition of the working-age population, with a larger portion of the population being unemployed [10, 14]. However, Katikireddi [21] showed that deteriorating mental health in England after the onset of the 2008 recession was not only the result of an increase in unemployment, but that it occurred as well among those in employment, which is coincident with our results, especially in the case among men. Among the possible hypothesis to explain this finding, the unstable labour market and the consequent increase of the perception of insecurity about keeping the jobs, the acceptance of a decline in working and employment conditions, and the consequences of the implementation of the labor-market reform in 2012 in Spain could be playing a role [31]. The different gender pattern observed in the employed population could be related to men’s lower satisfaction with their working environment and conditions, including their salary, as well as a higher fear to lose their job, compared to women in 2013 [45]. In contrast, being unemployed seems to have a more severe effect on women’s mental health. This might be because the life conditions of unemployed women seem to be worse than those of men, due in part to the fact that the unemployment coverage rate is lower for women [46], and their risk of being below the poverty line is higher in the Basque Country [47]. Regarding social inequalities, a similar gender pattern was found by Bartoll et al. who reported that in Spain, inequalities increased among men but remained stable among women [14]. However, a variety of results have been reported regarding changes in the equity patterns during times of crisis in different countries [7].

An important finding of this study is the relevance of age to understand the impact of the recession on mental health. As shown also by others (14,22,24], the 35–44 middle age population were most affected. Moreover, a clear increase of poor mental health risk among the student population was observed, which could be well related to feelings of pessimism about the future because of poor prospects in the current labour market. This same finding was shown in a Swedish study during the late 80’s and 90’s [28], but there is scarce evidence available for the current recession, limited to a study in Iceland describing an increased risk of high stress levels among female students from 2007 to 2009 [43] and a comparative study showing the deterioration of male students’ mental health until 2012 in some European countries [22]. Further research is therefore urged, especially in southern European countries such as Spain where the unemployment rates for young people is above 50 % and confidence about short-term future economic opportunities is at its lowest level among the young population [48]. On the other hand, according to our data, it seems that the crisis is not having a great impact on the mental health of the retired population. This could be due to the fact that they have become a relatively privileged social group as they are not losing disposable income to the same extent as other groups, and, unlike the rest of the population, their risk of poverty continues to diminish. The fact is that in Spain, many pensioners are currently maintaining whole families who have no other income [49].

While these results show a negative impact of the economic crisis on mental health, we need to be careful not to underestimate the true extent of the effects, since the full repercussions of recessions become evident only after many years. In Spain, it was not until after 2011 that the harshest austerity policies and financial cuts were implemented [50] and, therefore, we will have to wait some time before the full impact of unemployment and other adverse circumstances of this crisis can be analysed, especially among the disadvantaged sectors of the population [51]. Moreover, the welfare state regime type and the specific social insurance programmes, such as the extent of the unemployment insurance coverage, have been shown to account for an important part of nation-level variation in health [42] and health inequalities [52]. Long-term analyses of the impact of the Great Recession on mental health, therefore, should take these differential structural aspects into account.

## Conclusion

The main conclusion of the paper is that the Great Recession has been accompanied by a deterioration of the population mental health in the Basque Country, especially among in men. However, working status changes from the onset of the crisis, especially the increase of unemployment, cannot fully explain the observed changes in mental health. The deterioration of living conditions of economically inactive groups such as students, and of employment conditions of those who remain employed during the crisis may also be playing a role. Moreover, an increase of social class inequalities in mental among men health are shown.

It should be considered that most of the effects that austerity measures and financial cuts can be exerting on mental health were probably underestimated in the study, as these were especially harsh in Spain only after 2011. In consequence, public health practice should closely monitor the medium and long-term effects of the crisis as these may emerge only many years after the onset of the recessions.
